# Postprandial hyperglycemia as an etiological factor in vascular failure

**DOI:** 10.1186/1475-2840-8-23

**Published:** 2009-04-29

**Authors:** Koichi Node, Teruo Inoue

**Affiliations:** 1 Department of Cardiovascular and Renal Medicine, Saga University Faculty of Medicine, Saga, Japan

## Abstract

Postprandial hyperglycemia is characterized by hyperglycemic spikes that induce endothelial dysfunction, inflammatory reactions and oxidative stress, which may lead to progression of atherosclerosis and occurrence of cardiovascular events. Emerging data indicate that postprandial hyperglycemia or even impaired glucose tolerance may predispose to progression of atherosclerosis and cardiovascular events. There is evidence that postprandial hyperglycemia, but not fasting hyperglycemia, independently predicts the occurrence of cardiovascular events. We proposed a concept of 'vascular failure' as a comprehensive syndrome of vascular dysfunction extending from risk factors to advanced atherosclerotic disease. Postprandial hyperglycemia is therefore one of the very important pathophysiological states contributing to vascular failure. Accordingly, controlling postprandial hyperglycemia should be the focus of future clinical investigation as a potential target for preventing vascular failure.

## Introduction

Type 2 diabetes is associated with a markedly increased risk for atherosclerotic coronary arteries and cerebrovascular diseases [1,2]. In addition, there is evidence that abnormalities during the postprandial state, specifically postprandial hyperglycemia, are independent risk factors for atherosclerosis [3]. Recent epidemiological studies suggest postprandial hyperglycemia is an independent risk factor for cardiovascular disease that has effects greater than that of fasting hyperglycemia [4-6].

**Figure 1:**
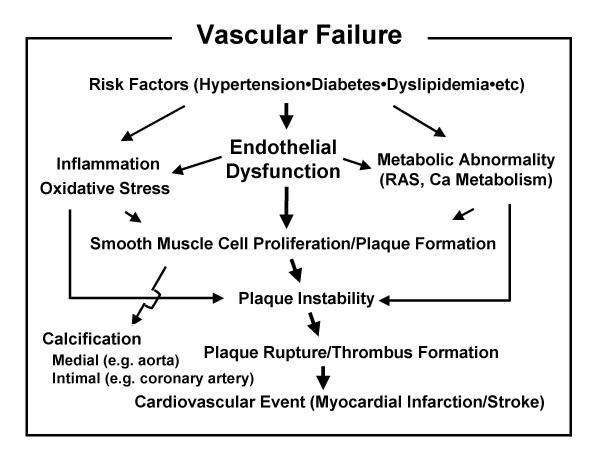
**Vasculular failure**. Vascular failure represents a comprehensive syndrome of vascular dysfunction that extends from risk factors to advanced atherosclerotic disease with arterial stenosis, leading ultimately to calcification of the vessel wall and serious vascular events caused by plaque rupture and thromboembolic occlusion. Calcification includes medial calcification, e.g., in aortic wall as result of diabetes and intimal calcification, e.g., in coronary artery.

**Figure 2:**
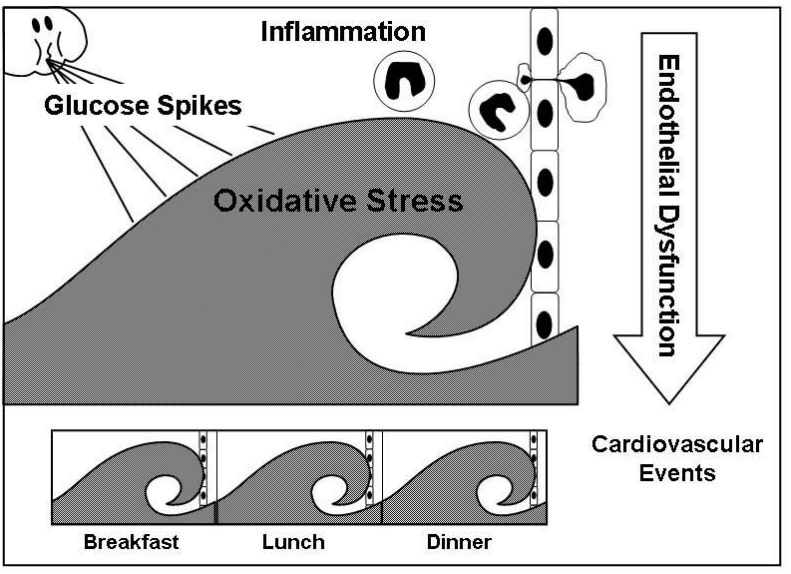
**Pathophysiology of postprandial hyperglycemia**. Hyperglycemic spikes following every meal induce oxidative stress, endothelial dysfunction and inflammatory reactions.

Atherosclerosis is a progressive disease characterized by the response of the vessel wall to chronic, multifactorial injury, which leads ultimately to the formation of atheromatous or fibrous plaques. Endothelial dysfunction is thought to be the initial stage of atherosclerosis. In addition to endothelial dysfunction, smooth muscle cell dysfunction metabolic abnormalities of the vessel wall including inflammation, oxidative stress and breakdown of neurohormonal balance occur in the early stage of the atherosclerosis process. We recently proposed a new concept termed 'vascular failure' that represents an integration of these vascular abnormalities [7]. Although Schwartz et al. formerly used the term 'vascular failure' as the failure of vascular remodeling response [8], our 'vascular failure' is distinguished from theirs and is defined as a comprehensive syndrome of vascular dysfunction extending from risk factors to advanced atherosclerotic disease with arterial stenosis, and finally to calcification of the vessel wall or serious vascular events caused by plaque rupture or thromboembolic occlusion (Fig. 1),

The pathophysiology of postprandial hyperglycemia is characterized by hyperglycemic spikes that induce oxidative stress [9], which in combination with soluble advanced glycation end products (AGEs) and lipid peroxidation products, act as key activators of upstream kinases, leading to endothelial dysfunction and expression of inflammatory genes [10] (Fig. 2). This article is an overview of the role of postprandial hyperglycemia, as a major fundamental disturbance, in the pathogenesis of vascular failure.

Recent evidence suggests that almost 2 of 3 patients with symptomatic cardiovascular disease have abnormal glucose homeostasis [11]. A significant number of these patients are not detected by increased fasting glucose levels, but rather by the presence of elevated glucose levels following a meal or during an oral glucose tolerance test [12]. Glucose intolerance is generally established by a 75 g glucose load test, with impaired glucose tolerance, or prediabetes, defined as a 2-hour postload glucose level of 140 to 200 mg/dl and overt type 2 diabetes as a postload glucose level>200 mg/dl. Postprandial hyperglycemia often occurs, even in the setting of good diabetic control assessed by hemoglobin A1c (HbA1c) and fasting glucose levels [13]. Population studies have shown that a fasting glucose level as low as 90 mg/dl may be associated with a 2-hour postprandial glucose level >200 mg/dl [14,15]. In the early stages of type 2 diabetes, even when fasting glucose and HbA1c are within nornal ranges, postprandial hyperglycemia causes macrovascular complications such as myocardial infarction or stroke as well as microvascular complications [12,14,16,17]. Emerging data indicate that even impaired glucose tolerance may predispose to progression of atherosclerosis and cardiovascular events [12]. There is evidence that postprandial hyperglycemia, but not fasting hyperglycemia, independently predicts the occurrence of cardiovascular events [16]. For example, the Funagata Diabetes Study showed consistently that 1- or 2-hour postload glucose levels were better predictors of cardiovascular risk than either fasting glucose or HbA1c levels [14], while the Diabetic Epidemiology: Collaborative Analysis of Diagnosis Criteria in Europe (DECODE) study demonstrated a continuous graded and direct relationship between 2-hour postload glucose levels and risk for cardiovascular death [18]. Even in patients classified as having normal glucose tolerance with a postload glucose <140 mg/dl, the level of postload glycemia correlates with the risk of cardiovascular death and all-cause mortality [19]. The risk for postload glycemia begins to increase at levels >80 mg/dl and by 140 mg/dl, the point at which patients are traditionally classified as having impaired glucose tolerance (IGT) or prediabetes, cardiovascular risk is already increased by 58%.

It is now well recognized that endothelial dysfunction is the initial stage in the development of atherosclerosis [20]. Endothelial dysfunction is characterized by a reduction in the production and bioavailability of endothelium-derived relaxing factors, in particular, nitric oxide (NO), generated from L-arginine by endothelial NO synthase (eNOS). A reduction in NO bioavailability leads to impaired endothelium-dependent vasodilation, the functional manifestation of endothelial dysfunction [21]. On the other hand, it also comprises a specific state of "endothelial activation", characterized by a proinflammatory, proliferative and procoagulation milieu that enhances all the various stages of atherogenesis [22]. Given this relationship between endothelial dysfunction and atherosclerosis, it is likely that the status of endothelial function may reflect the propensity of an individual to develop atherosclerotic disease. The presence of endothelial dysfunction therefore serves as an early marker of vascular failure.

**Figure 3:**
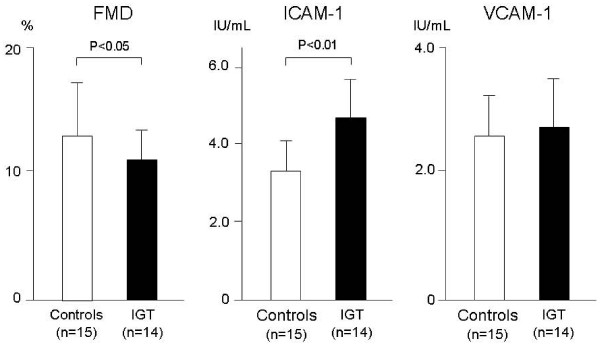
**Markers of endothelial function and inflammation in patients with impaired glucose tolerance and subjects with normal glucose tolerance (control)**. Compared to control subjects, patients with IGT have reduced flow mediated vasodilation and higher circulating levels of ICAM-1, but not VCAM-1.

Endothelial function is impaired not only in patients with diabetes but also in patients with IGT. We observed flow-mediated vasodilation (FMD) was reduced in patients with IGT, compared to subjects with normal glucose tolerance (Fig. 3). An acute increase in glycemia affects endothelial function, with both *in vitro *and *in vivo *studies confirming this direct role of hyperglycemia. The presence of high glucose concentrations *in vitro *reduces acetylcholine-induced endothelium-dependent vasodilation in a concentration-dependent manner, with the effect being obtained simply by exposing the preparations to high glucose levels [23]. *In vivo *studies have also demonstrated that hyperglycemic spikes induce endothelial dysfunction [24,25]. Williams et al. [26], using FMD, showed that acute hyperglycemia induced in healthy subjects by intra-arterial infusion of 50% dextrose resulted in impaired endothelium-dependent vasodilation. This finding indicates that elevated glucose levels are a major cause of endothelial dysfunction associated with diabetes mellitus. Shige et al. [27] also demonstrated FMD was attenuated after the intake of fat- and sucrose-rich meals in patients with type 2 diabetes. They showed both the level and change in postprandial FMD correlated significantly with postprandial changes in blood glucose levels, thereby establishing postprandial hyperglycemia as a determinant of reduced flow-mediated vasodilation.

These effects of hyperglycemia are probably linked with reduced production and/or bioavailability of NO, as hyperglycemia-induced endothelial dysfunction is counterbalanced by increased production of arginine [28]. The inter-relationship between NO production and bioavailability has to be fully understood, because it is not yet clear whether hyperglycemia reduces production of NO, or more probably, increases NO production in association with significant increases in the levels of its inhibitor, the superoxide anion, resulting in a reduction in NO bioavailability [29]. Furthermore, a rapid decrease in FMD has been demonstrated in patients with IGT during an OGTT that correlated with the degree of glycemia measured at 2 hours [25].

Recent data suggest that AGEs also have a role in the development of endothelial dysfunction [30]. AGEs are a heterogeneous group of moieties, one of the most representative being carboxymethyllysine (CML). Diet is a major source of exogenous AGEs, with food AGE content being highly dependent on food nutrient composition, as well as the method, temperature and duration of cooking [31]. Approximately 10% of ingested AGEs are absorbed rapidly and partly retained in the body, where they exert a variety of pathological effects [32] including binding and activation of AGE receptors [33]. AGE precursors such as methylglyoxal (MG) also activate AGE receptors, with an *in vivo *study demonstrating endogenous MG synthesis increased in parallel with hyperglycemia [34]. In the post-prandial period, absorbed and endogenously generated AGEs and MG act synergistically to decrease vascular function by directly scavenging NO and increasing oxidative stress.

## Inflammation in postprandial hyperglycemia

It is well established that inflammation is a major factor in the pathogenesis of vascular failure. Inflammation in diabetes has long been assessed and considered by the measurement of various inflammatory markers such as high-sensitivity C reactive protein (hsCRP). Various inflammatory cells and mediators play an essential role in the inflammatory process, with initiation and progression of atherosclerosis being characterized by recruitment of leukocytes such as monocytes and T-lymphocytes to the endothelium of the artery wall. Adhesion molecules regulate this interaction between the endothelium and leukocytes [35]. Of the various adhesion molecules, intracellular adhesion molecule (ICAM)-1 and vascular cell adhesion molecule (VCAM)-1 have attracted particular interest. An increase in the circulating forms of these molecules has been demonstrated in subjects with vascular disease [36] and also in subjects with diabetes, with or without vascular disease [37,38]. We observed that circulating levels of ICAM-1, but not VCAM-1, were higher even in patients with IGT, compared to healthy subjects (Fig. 3). In addition, it has been demonstrated that acute hyperglycemia in both normal and diabetic subjects is a sufficient stimulus to increase circulating levels of ICAM-1, thereby activating one of the first stages of the atherogenic process [39,40]. The concept of atherosclerosis as an inflammatory disease, even in diabetes, is now well established [41], with evidence that acute hyperglycemia during either a hyperglycemic clamp [42] or the postprandial state [43] increases production of plasma interleukin-6, tumor necrosis factor-α and interleukin-18.

Although previous research has focused on monocytes and T-lymphocytes as the predominant types of inflammatory cells involved in atherosclerosis [44,45], there is growing evidence that neutrophil activation is also a major participant in the inflammatory process of ischemic cardiovascular disease, particularly during the acute inflammatory response. In addition, of the various types of leukocytes, the neutrophil count was shown to be the best predictor of future cardiovascular events in a large patient cohort with a high risk of coronary artery and cerebrovascular diseases [45]. Peripheral neutrophils are the most important productive source of reactive oxygen specimens. Activated neutrophils contribute markedly to oxidative stress and inflammation in type 2 diabetes, which subsequently evolves causing angiopathy and atherosclerosis [46]. An adhesion molecule, β2-integrin Mac-1 (CD11b/CD18), is essential for firm adhesion of both neutrophils and monocytes to vascular endothelial cells. Mac-1 binds to endothelial surface ICAM-1 [47] and also to platelets by binding to either fibrinogen or several platelet receptors such as glycoprotein (GP) Ibα [48] and ICAM-2 [49]. Clinical studies have shown that Mac-1 is activated and upregulated on the surface of neutrophils localized in injured vessels following coronary angioplasty [50]. Upregulation of Mac-1 on the surface of neutrophils has also been observed in patients with type 2 diabetes [51]. In addition, it has been reported that agonist-induced ex-vivo upregulation of Mac-1 on the surface of neutrophils is enhanced in patients with diabetes [52]. We have previously observed expression of Mac-1 on the surface of isolated neutrophils using flowcytometric analysis before and 120 min after an oral 75 g glucose load in patients who had not been diagnosed with diabetes mellitus and whose fasting plasma glucose level was <126 mg/dl. Our results showed that compared to baseline values, Mac-1 was upregulated even on unstimulated neutrophils 120 min after the glucose load in patients with postprandial hyperglycemia, defined as a glucose level of ≥ 200 mg/dl at 120 min. In addition, upregulation of fMLP-induced Mac-1 was enhanced significantly at 120 min compared to baseline, not only in patients with postprandial hyperglycemia, but also in patients with IGT defined as a glucose level at 120 mim ≥ 140 mg/dl. However, these changes were not evident in patients with normal glucose tolerance defined as a glucose level <140 mg/dl at 120 min (Fig. 3). These results suggest that acute increases in plasma glucose may alter neutrophil function from inactivated to potentially activated forms in patients with postprandial hyperglycemia and impaired glucose tolerance.

## Contribution of postprandial hyperglycemia to oxidative stress

Oxidative stress defined as dysregulation of the cellular redox state, plays a pivotal role in the pathogenesis of vascular failure, especially vascular endothelial dysfunction [53]. The superoxide anion is formed by univalent reduction of molecular oxygen. Although several enzymes are involved in the generation of superoxide anions, including xanthine oxidase, NADH/NADPH oxidase, lipoxygenase and NOS, mitochondria are the major source of superoxide anion production *in vivo *[54]. Superoxide anions are reduced to hydrogen peroxide either spontaneously or by enzymatic catalyzed dismutation. Transition metal- catalyzed interactions between iron or copper and hydrogen peroxide produce highly toxic hydroxyl radicals. Recent studies demonstrate that hyperglycemia induces an overproduction of superoxide anions by the mitochondrial electron-transport chain [55]. Superoxide overproduction is accompanied by increased NO production, caused by an uncoupled state between eNOS and inducible NOS (iNOS), a phenomenon that favors the formation of the strong oxidant, peroxynitrite, which in turn damages DNA. DNA damage is an obligatory stimulus for activation of the nuclear enzyme poly(ADP-ribose) polymerase. Poly(ADP-ribose) polymerase activation, in turn, depletes the intracellular concentration of its substrate NAD+, resulting in a decreased rate of glycolysis, electron transport and adenosine triphosphate formation, leading to adenosine diphospate-ribosylation of reduced glyceraldehyde-phosphate dehydrogenase [56]. These processes result in acute endothelial dysfunction in diabetic blood vessels that contributes overtly to the development of cardiovascular disease. There is both indirect and direct evidence to support the concept that acute hyperglycemia promotes the development of cardiovascular disease in subjects with IGT through the production of oxidative stress. The direct evidence is based on the effects of postprandial hyperglycemia on oxidative stress markers such as nitrotyrosine and 8-iso-prostaglandin F_2α _(8-iso-PGF_2α_).

Oxygen free radicals react with nitric oxide to form peroxynitrite, a powerful oxidant that may directly oxidize proteins, lipids, and DNA through a nitronium-like intermediate, resulting in formation of carbonyls from side-chain and peptide-bond cleavage [57]. Peroxynitrite has an affinity for tyrosine residues, with this reaction producing nitrotyrosine [58]. Evidence from several studies supports a direct role of hyperglycemia promoting over-generation of nitrotyrosine. For example, nitrotyrosine formation has been detected in the artery walls of monkeys during hyperglycemia [59], in the plasma of healthy subjects during hyperglycemic clamps [60] or OGTTs [61] and also in patients with diabetes mellitus during an increase in postprandial hyperglycemia, with nitrotyrosine production being dependent on the level of glycemia [62]. Hyperglycemia was also observed to be accompanied by nitrotyrosine deposition in perfused working hearts of rats, that appeared to be related to unbalanced production of NO and superoxide anion caused by over-expression of iNOS [63]. Nitrotyrosine formation has been shown to be followed by the development of endothelial dysfunction in both healthy subjects [60] and in the coronary arteries of perfused hearts [63]. This effect is not surprising, as nitrotyrosine has been shown to be directly harmful to endothelial cells [64].

Determination of specific isoprostane isomers such as 8-iso-PGF_2α _in urine have been proposed as markers of oxidative stress. Isoprostane is formed collectively from free radical-mediated oxidation of arachidonic acid [65]. As this fatty acid is distributed ubiquitously in cell membranes, measurement of urinary isoprostanes most likely provides an excellent reflection of oxidative stress in the whole body. Several studies have demonstrated hyperglycemia is associated with increased rate of formation and urinary excretion rate of 8-iso-PGF_2α _[66]. The urinary excretion rate of 8-iso-PGF_2α _has been reported to be increased significantly in patients with type 2 diabetes patients compared with age-matched healthy subjects. Furthermore, a significant correlation was observed between blood glucose and urinary 8-iso-PGF_2α_, suggesting that an increase in oxidative stress may be related, at least in part, to determinants of diabetic control. Such results are consistent with *in vitro *findings of enhanced formation and release of 8-iso-PGF_2α _by porcine vascular smooth muscle cells cultured under hyperglycemic conditions [67]. In a recent study, Monnier et al. [68] demonstrated a strong positive correlation between the urinary excretion rate of 8-iso-PGF_2α _and glycemic variability assessed by the mean amplitude of glycemic excursions (MAGE). A statistically significant correlation was also observed with the mean postprandial glucose increment, although this relationship was not as strong. These findings indicate that the triggering effect of acute glycemic excursions on oxidative stress should be integrated into glycemic disorders as they are much broader than acute postprandial spikes. As a consequence, the concept that postprandial hyperglycemic spikes are "dangerous waves" should be extended to both upward (postprandial) and downward (interprandial) acute fluctuations of glucose levels around a mean value.

## Therapeutic Paradigm

A number of clinical trials have demonstrated that specific pharmacological approaches can reduce the impact of postprandial glycemic excursions on overall glycemic control [69-71]. However, to date, there have been no prospective clinical trials evaluating the impact of improved postprandial hyperglycemia on long-term outcomes in patients with diabetes. Given recent data on the impact of postprandial glycemia on overall glucose control [72], it is likely that therapeutic approaches that focus on this aspect of overall glycemia will benefit patients in the long term. Traditional diabetic agents such as insulin and sulfonylureas predominantly lower fasting glucose and are less effective at reducing postprandial hyperglycemia. Although sulfonylureas target insulin secretion directly through the beta cell potassium channel, they may also have an impact on fasting and postprandial glycemia. However, the pharmacokinetics of the majority of these agents are not tailored toward acute insulin release, and therefore they do not correct abnormalities in early-phase insulin secretion [73]. On the other hand, the pharmacokinetics and mechanisms of action of several agents are directed specifically at postprandial hyperglycemia. Such agents include the meglitinide class of drugs, α-glucosidase inhibitors and thiazolidinediones.

The meglitinide analogues include repaglinide, nateglinide and mitiglinide, which are modern nonsulfonylurea secretagogues that restore the first-phase insulin response. A single dose of nateglinide has been shown to reduce postprandial endothelial dysfunction by lowering postload glycemia in patients with type 2 diabetes [74]. In a randomized trial of diabetic patients, repaglinide reduced postprandial glucose levels to a greater degree than the sulfonylurea, glyburide, whereas glyburide was more effective for lowering fasting glucose levels. Despite identical reductions in HbA1c levels in the 2 groups, repaglinide caused regression of carotid intima-media thickness in 52% of patients, compared with only 18% of patients treated with glyburide [75]. This regression in atherosclerosis was shown to be directly proportional to the reduction in postprandial hyperglycemia.

**Figure 4:**
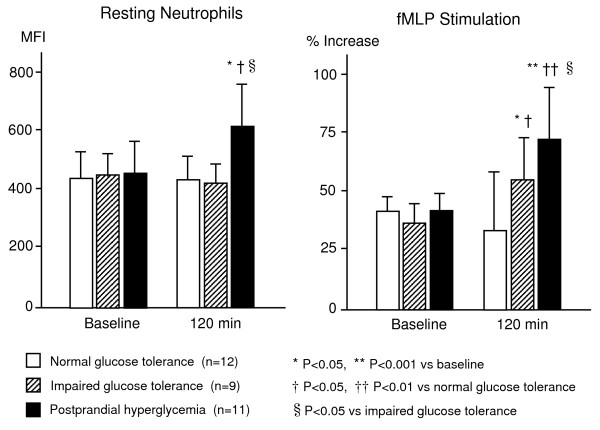
**Expression of β2 integrin Mac-1 on the surface of isolated neutrophils before and 120 min after an oral 75 g glucose load in 11 patients with postprandial hyperglycemia (fasting plasma glucose level <126 mg/dl and glucose level at 120 min ≥ 200 mg/dl), 9 patients with impaired glucose tolerance (IGT) (fasting plasma glucose level <126 mg/dl and glucose level at 120 min ≥ 140 mg/dl) and 12 patients with normal glucose tolerance (fasting plasma glucose level <126 mg/dl and glucose level at 120 min <140 mg/dl)**. In patients with postprandial hyperglycemia, Mac-1 expression on unstimulated neutrophils was upregulated at 120 min after glucose loading compared to baseline. In addition, fMLP-induced upregulation of Mac-1 was enhanced significantly at 120 min in both patients with postprandial hyperglycemia and patients with IGT. In contrast, these changes were not evident in patients with normal glucose tolerance.

**Figure 5:**
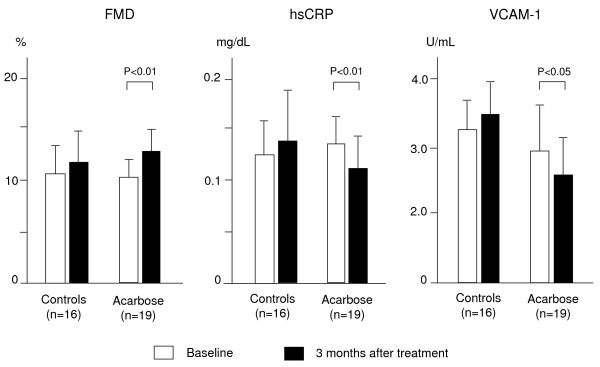
**Effects of acarbose on vascular endothelial function and inflammatory status in patients with mild diabetes mellitus**. We compared flow mediated vasodilation (FMD) and levels of high sensitivity C reactive protein (hsCRP) and circulating vascular cell adhesion molecule (VCAM)-1 in 16 patients undergoing dietary therapy alone (controls) and 19 patients receiving acarbose 100 mg 3 times daily before each meal. FMD increased and hsCRP and VCAM-1 levels decreased after 3 months of acarbose treatment, although these effects were absent in the controls.

**Figure 6:**
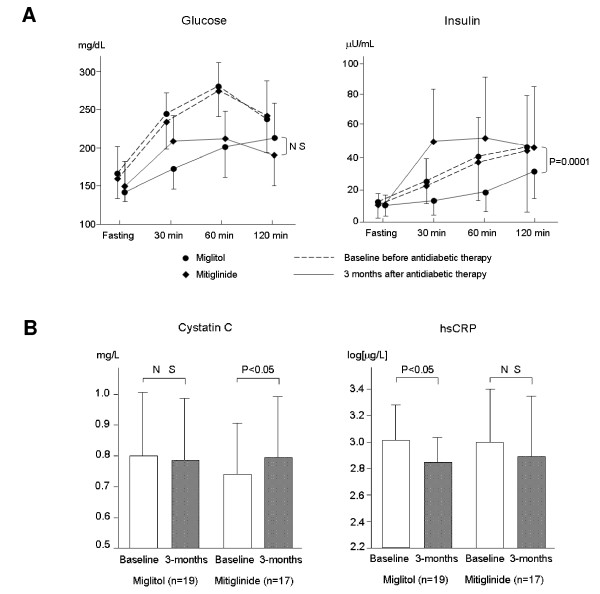
**Comparison of the effects of miglitol and mitiglinide on postprandial glucose and insulin metabolism after meal tolerance testing in patients with mild diabetes mellitus**. A pre-specified breakfast was prepared, containing 63.8 g of carbohydrate, 24.6 g of protein, 11.0 g of fat, 1.2 g of sodium and a total of 466 calories. Three months of administration of both agents caused similar improvements in postprandial hyperglycemia, although different patterns of insulin secretion were observed (A). Serum cystatin C levels did not change in the miglitol group, although the levels increased in the mitiglinide group. Miglitol caused a significant decrease in hsCRP levels whereas mitiglinide did not (B).

Alpha-glucosidase inhibitors block the α-glucosidase enzyme, thereby slowing the rate of carbohydrate digestion including starches and disaccharides. In diabetic or glucose-intolerant patients, α-glucosidase inhibitors lower postload glucose peaks by between 30 to 70 mg/dl and reduce HbA1c by about 0.7%, with negligible effects on fasting glucose levels [76]. Long-term acarbose therapy also reduces the levels of triglycerides and chylomicrons, possibly by improving insulin sensitivity [77]. A recent study showed that even a single mixed meal of 450 calories caused substantial and immediate deterioration in endothelial function in diet-treated patients with diabetes [76]. Pretreatment of these same subjects with a single dose of acarbose markedly reduced postprandial hyperglycemia and associated endothelial dysfunction after the ensuing meal. The Study to Prevent Non-Insulin-Dependent Diabetes Mellitus (STOP-NIDDM) was an international trial of 1,429 subjects with impaired glucose tolerance who were randomized to either acarbose 100 mg, 3 times daily with meals or placebo [78]. After 3.3 years, acarbose significantly reduced the primary end point of the study, the progression to new diabetes, by 25%. In this trial, acarbose treatment was associated with significant reductions of 49% for any cardiovascular event and 91% for myocardial infarction. Acarbose also slowed progression of carotid atherosclerosis and reduced the development of new hypertension by 34%, results that indicated postprandial dysmetabolism may play a role in the genesis of hypertension. A large retrospective meta-analysis of seven long-term studies of acarbose in patients with type 2 diabetes showed significant risk reductions of 35% for cardiac events and 64% for myocardial infarction [79]. We have also previously observed increases in FMD and a reduction in hsCRP and VCAM-1 levels after 3 months of treatment with acarbose (Fig. 4). This suggests attenuation of postprandial dysmetabolism induced by acarbose results in improved endothelial function and inflammatory status, possibly slowing the progression of atherosclerosis. In addition, in a recent study in patients with mild diabetes mellitus we used meal tolerance testing to compare the effects of a new α-glucosidase inhibitor, miglitol, with those of a meglitinide analogue, mitiglinide, on postprandial glucose and insulin metabolism. Other glucose metabolism-related markers, atherosclerosis-related markers and renal function were also assessed. We showed that three months of administration of both agents caused similar improvements in postprandial hyperglycemia, although different postprandial patterns of insulin secretion were observed (Fig. 5A). The changes in 1, 5-anhydroglucitol levels after 3 months were significantly higher in the miglitol group than in the mitiglinide group. Insulin resistance assessed by the homeostasis model assessment index and urinary albumin excretion decreased significantly in the miglitol group but not in the mitiglinide group. Serum cystatin C levels did not change in the miglitol group, although the levels increased in the mitiglinide group. Miglitol caused a significant decrease in hsCRP levels, whereas mitiglinide did not (Fig. 5B and Fig 6), while serum adiponectin levels were increased significantly only by miglitol therapy [80,81]. These results suggest that miglitol has anti-inflammatory and renoprotective effects, possibly associated with an improvement in insulin resistance.

The thiazolidinedione peroxisome proliferator-activated receptor-γ (PPAR-γ) activating class of drugs, such as troglitazone, rosiglitazone or pioglitazone, are insulin-sensitizing agents and are not generally considered for their effects on insulin secretion [82]. However, as demonstrated with troglitazone, this class of compounds, when used in subjects with impaired glucose tolerance, may improve glucose-coupled insulin secretion and reduce the level of postprandial hyperglycemia [83]. PPAR-γ agonism has been shown to have anti-inflammatory effects and to improve endothelial function by regulating the cellular redox state. Thiazolidinediones have also been shown to decrease inflammatory markers such as hsCRP [84] and to improve FMD of the brachial artery [85]. In a large randomized clinical trial of pioglitazone in 5238 patients with type 2 diabetes with macrovascular disease there was some evidence of a reduction in adverse cardiovascular outcomes compared to matching placebo, when the treatments were taken in combination with glucose-lowering drugs and other medications [86]. Pioglitazone was also reported to reduce the rate of progression of carotid intimal medial thickness [87] and coronary atherosclerosis assessed by intravascular ultrasound imaging [88], when compared to glimepiride.

Several new drugs with glucose-lowering actions that may offer certain clinical advantages have recently become available. These include injectable glucagon-like peptide-1 (GLP-1) receptor agonists and oral dipeptidyl peptidase-4 (DPP-4) inhibitors. GLP-1 receptor agonists, such as exenatide, stimulate nutrient-induced insulin secretion and reduce inappropriate glucagon secretion, whilst delaying gastric emptying and reducing appetite. These agents have a low risk of hypoglycaemia in combination with sustained weight loss. The DPP-4 inhibitors, sitagliptin and vildagliptin, are generally weight neutral, and have less marked gastrointestinal adverse effects than the GLP-1 receptor agonists. Vildagliptin is generally well tolerated whether administered alone or in combination with glyburide or pioglitazone, and is not associated with hypoglycemia. Co-administration of vildagliptin with either glyburide or pioglitazone in patients with type 2 diabetes improves postprandial glycemic control without notable effects on drug pharmacokinetics [89].

## Conclusion

During postprandial hyperglycemia, hyperglycemic spikes induce endothelial dysfunction, inflammatory reactions and oxidative stress, which may lead to progression of atherosclerosis and occurrence of cardiovascular events. Epidemiological and mechanistic data suggest that postprandial hyperglycemia or even IGT may play a role in the development and progression of atherosclerotic disease, as suggested by preliminary evidence that controlling postprandial hyperglycemia reduces the incidence of myocardial infarction in people with IGT [78]. Postprandial hyperglycemia is therefore one of the very important pathophysiological states contributing to vascular failure. Accordingly, controlling postprandial hyperglycemia should be the focus of future clinical investigation as a potential target for preventing vascular failure.

## Competing interests

The authors declare that they have no competing interests.

## Authors' contributions

KN was responsible for the study design of endothelial function and inflammatory markers after treatment with acarbose and final approval of this manuscript. TI was responsible for the study design of neutrophil activation using flowcytomeric analysis during glucose load, literature search, and writing the review article.
